# Disease-specific health related quality of life patient reported outcome measures in Genodermatoses: a systematic review and critical evaluation

**DOI:** 10.1186/s13023-017-0739-5

**Published:** 2017-12-29

**Authors:** John W. Frew, Mark Davidson, Dedee F. Murrell

**Affiliations:** 1 Department of Dermatology, Liverpool Hospital, Sydney, Australia; 2 Faculty of Medicine, UNSW, Sydney, Australia; 3 Prince of Wales Hospital, Sydney, Australia; 4 Department of Dermatology St George Hospital, Sydney, Australia

**Keywords:** Quality of life, Patient reported outcome measures, Genodermatoses, COSMIN checklist, Measurement properties

## Abstract

**Background:**

Health Related Quality of Life (HR-QoL) Patient reported outcome measures (PROMs) have high utility in evaluation of new interventions in genodermatoses, however inconsistent standards of development and validation have hampered widespread acceptance and adoption.

**Objectives:**

To identify all published HR-QoL PROMs in genodermatoses and critically evaluate their development and measurement properties.

**Methods:**

This systematic review was registered with PROSPERO (CRD42016053301). Ovid Medline, Embase and PsycINFO databases were utilised for literature review using predefined inclusion and exclusion criteria. PROM development was assessed using the COSMIN Checklist and measurement properties were assessed against quality criteria for measurement properties of health standard questionnaires.

**Results:**

15 HRQoL PROMs in genodermatoses were identified. Major areas of deficiency in development were internal consistency, reliability and structural validity. No PROM satisfied measurement property standards for agreement, responsiveness or floor and ceiling effects. Four PROMs included Minimal Important Change scores for interpretability. Issues regarding the generalisability of the evaluated PROMs in culturally diverse and paediatric populations remain unresolved.

**Conclusions:**

The overall standards of development and measurement properties in PROMs in genodermatoses is fair, despite no single instrument meeting all requirements. None are perfectly validated according to COSMIN criteria but seven of the fifteen PROMs may be appropriate pending further validation. The development of culturally appropriate and child-specific variants of PROMs should be a priority in order to increase the utility of patient based outcome measures in genodermatoses in various patient populations.

**Electronic supplementary material:**

The online version of this article (10.1186/s13023-017-0739-5) contains supplementary material, which is available to authorized users.

## Background

Health related Quality of life (HR-QoL) refers to the physical, psychological and social domains of health that are influenced by a person’s experiences, beliefs, expectations and perceptions. [1, 2]. Patient reported outcome measures (PROMs) assessing HR-QoL are increasingly used in dermatology to assess the impact of disease upon individuals and carers and has found utility as a method of understanding chronic lifelong disease and impairment [2, 3]. The significant psychosocial impact of dermatological disease can also result in disconnect between clinical manifestations and burden of disease [2, 3]. HR-QoL PROMs are responsive to changes in disease activity [4] and hence have high utility in assessing new therapeutic interventions, particularly when objective biochemical markers of disease activity are unknown. However, a lack of awareness of the standardised methodologies in the development of PROMs hampers widespread adoption [4–6].

Methodologies and nomenclature regarding the development and validation of HR-QoL PROMs are well established and standardised [4–6] (Table 1), with ‘COnsensus based Standards for selection of health Measurement INstruments’ (COSMIN) checklist [7, 8] and ‘quality criteria for measurement properties of health standard questionnaires’ [9] enabling objective evaluation of existing HR-QoL PROMs.

**Table 1:** Definitions of Important Measurement Properties: Comparison of the COSMIN Taxonomy and Definitions and Quality Criteria for Measurement Properties Definitions [7–9]

Measurement Property	COSMIN Definition [7, 8]	Quality Criteria for Measurement Properties Definition [9]
Content Validity	The degree to which the content of an HR-PRO instrument is an adequate reflection of the construct to be measured	The extent to which the domain of interest is comprehensively sampled by the items in the questionnaire
Internal Consistency	The degree of the interrelatedness among the items	The extent to which items in a (sub)scale are intercorrelated, thus measuring the same construct
Criterion Validity	The degree to which the scores of an HR-PRO instrument are an adequate reflection of a ‘gold standard’	The extent to which scores on a particular questionnaire relate to a gold standard
Construct Validity	The degree to which the scores of an HR-PRO instrument are consistent with hypotheses (for instance with regard to internal relationships, relationships to scores of other instruments, or differences between relevant groups) based on the assumption that the HRPRO instrument validly measures the construct to be measured	The extent to which scores on a particular questionnaire relate to other measures in a manner that is consistent with theoretically derived hypotheses concerning the concepts that are being measured
Structural Validity (Aspect of Construct Validity)	The degree to which the scores of an HR-PRO instrument are an adequate reflection of the dimensionality of the construct to be measured	
Hypothesis Testing (Aspect of Construct Validity)	Item construct validity	
Cross Cultural Validity (Aspect of Construct Validity)	The degree to which the performance of the items on a translated or culturally adapted HR-PRO instrument are an adequate reflection of the performance of the items of the original version of the HR-PRO instrument	
Reproducibility		
Agreement	The systematic and random error of a patient’s score that is not attributed to true changes in the construct to be measured	The extent to which the scores on repeated measures are close to each other (absolute measurement error)
Reliability	The proportion of the total variance in the measurements which is due to ‘true’† differences between patients	The extent to which patients can be distinguished from each other, despite measurement errors (relative measurement error)
Responsiveness	The ability of an HR-PRO instrument to detect change over time in the construct to be measured	The ability of a questionnaire to detect clinically important changes over time
Floor and Ceiling Effects	(Not Defined)	The number of respondents who achieved the lowest or highest possible score
Interpretability	Interpretability is the degree to which one can assign qualitative meaning - that is, clinical or commonly understood connotations – to an instrument’s quantitative scores or change in scores.	The degree to which one can assign qualitative meaning to quantitative scores

Disease-specific PROMs are useful in complex conditions such as genodermatoses where the content validity of generic PROMs is questionable [4, 5]. Sub-optimal methodologies in development of disease-specific PROMs may cause cascading validity issues surrounding the use of PROMs as outcomes in clinical trials, with consequent risk of recommending the use of an intervention with no significant benefit, or denying recommendation to a treatment with significant benefit to a subgroup of individuals. This is especially pertinent in genodermatoses where interventions and therapies (aside from supportive measures) are often limited [2]. Whilst generic PROMs are more accessible for use in patients with genodermatoses, their validity is questionable, particularly in the setting of severe genetic skin disease [2]. As generic dermatology PROMs are validated in the general dermatology population, when they are employed in genodermatoses cohorts, they suffer from suboptimal content validity, floor and ceiling effects, limited responsiveness and variability in utility between different genodermatoses [2, 5, 6]. Critical evaluation and recommendation of generic HR-QOL PROMs for use in genodermatoses cohorts is beyond the scope of this current review.

Currently, no critical evaluation of existing disease-specific PROMs in genodermatoses has been undertaken. Critical evaluation and recommendation of disease-specific HR-QOL PROMs in genodermatoses would aid in upholding quality standards in HR-QOL measurement in genodermatoses through identifying appropriate measures and increasing awareness of existing validity and reliability standards.

## Aims

This systematic review aims to identify all published disease-specific HR-QoL PROMs in genodermatoses. Each PROM will be critically evaluated using the COSMIN Checklist [7, 8] and quality criteria for measurement properties of health standard questionnaires [9] to assess adherence to the current standards of development and validation. A brief overview of previous use of these PROMs and general recommendations for their use will be made adapting the published criteria for HR-QoL PROMs in Atopic Dermatitis by Schmitt et al. [10] providing clear recommendation for the clinical use of HR-QOL in genodermatoses.

## Methods

This systematic review was registered with PROSPERO (CRD42016053301) [11].

Ovid Medline, ‘Epub ahead of print and Non-indexed citations’ Embase and PsycINFO databases were searched to identify HR-QoL PROMs in genodermatoses. A full search strategy is included in Additional file 1: Figure S1. All articles were screened for eligibility with pre-determined inclusion and exclusion criteria (Table 2) by 2 independent authors (JF, MD) with any disagreements mediated by a third author (DM) with inclusion or exclusion decided by simple majority.

**Table 2:** Inclusion and Exclusion Criteria for this Review

Inclusion Criteria:
Disease is a Monogenic Inherited Disorder AND Disease is Primarily Dermatological in Nature AND Study Pertains to Qualitative or Quantitative Studies of HR-QoL OR Study Pertains to Development and/or Validation Studies of HR-QoL PROM OR Study Pertains to Assessment of HR-QoL in non-disease-affected carers and family members
Exclusion Criteria
Studies not investigating HR-QoL as a major outcome OR Qualitative studies without a pre-defined qualitative methodology OR Development and Validation studies of disease severity or functional outcomes without HR-QoL assessment.

Evaluation of the studies describing the identified PROMs as well as their measurement properties were performed using the COSMIN Checklist [7, 8] and the ‘quality criteria for measurement properties of health standard questionnaires’ by Terwee et al. [9] respectively. The ‘Worst score counts’ method was utilised for overall assessment of each methodological quality [8]. Selection bias and recall bias of included studies was addressed in relevant sections of the COSMIN checklist [7, 8]. The evaluation of measurement properties was summarised into an overall recommendation, (graded A-D) derived from published criteria by Schmitt et al. [10] based upon OMERACT filter 2.0. Assessment was undertaken by two independent authors (JF, MD) evaluated each PROM, collated and compared results, with any disagreements mediated by referral to a third author (DF) with final decision made by simple majority. Author JF was excluded from evaluation of one of the PROMs (QOLEB [12]) due to a conflict of interest in being involved in the development of this PROM. As per the COSMIN manual [7, 8], criterion validity does not usually apply to HR-QOL related PROMs. The only exception would occur when “a shortened instrument is compared to the original long version” [7, 8]. Hence COSMIN assessment of criterion validity was not considered appropriate.

All results and discussion pertaining to the COSMIN checklist will adhere to definitions as defined by the COSMIN initiative [7, 8]. All results and discussions pertaining to the ‘quality criteria for measurement properties of health standard questionnaires’ by Terwee et al. [9] will adhere to definitions as per Terwee et al. [9]. Comparison of these definitions is provided in Table 1.

## Results

**Fig. 1:**
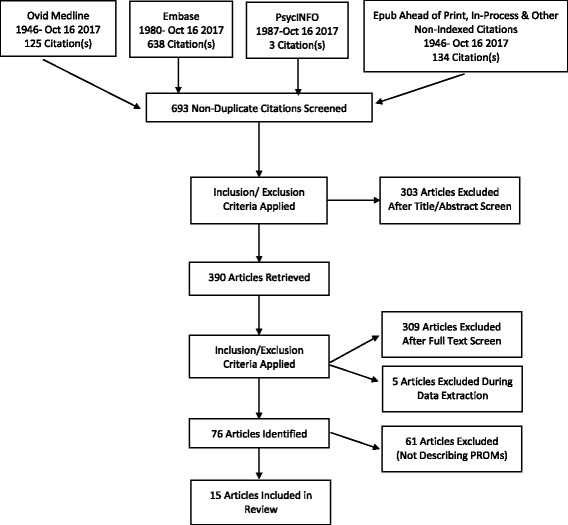
Flow Diagram of Literature Search and Systematic Review

The PRIMSA flow diagram is presented in Fig. 1. Of 693 non-duplicate citations, 303 were removed after screening with eligibility criteria (Table 2). 390 full text articles were assessed, with 309 articles excluded in line with eligibility criteria. 5 articles pertaining to 2 conditions (Osteogenesis Imperfecta and Chronic Granulomatous Disease) were excluded during data extraction due to these conditions not being primarily dermatological in nature. 76 articles (listed in Additional file 2: Figure S2) underwent full text screening by 2 independent authors (JF and MD) to identify 15 papers reporting the development of 15 disease-specific PROMs across 10 discrete genodermatoses. Genodermatoses included congenital ichthyoses [13, 14], pachyonychia congenita [15–17], darier’s disease [18], neurofibromatosis type 2 [19], hereditary haemorrhagic telangiectasia [20, 21], hereditary angioedema [22, 23], epidermolysis bullosa [12, 24], fabry disease [25], basal cell naevus syndrome [26, 27], and peutz -jeagher syndrome [28]. The remaining 61 papers employed generic PROMs or evaluated HR-QoL using the PROMs in the specific condition(s) as opposed to describing development and/or validation. Results of the COSMIN Checklist evaluation are presented in Table 3, with the results of evaluation using ‘quality criteria for measurement properties of health standard questionnaires’ are presented in Table 4.

**Table 3:** Critical evaluation of development of Disease-Specific PROM using COSMIN criteria examined in this review

Condition	HR-QoL PROM	Study	Items	CTT / IRT	Internal Consistency	Reliability	Measurement Error	Content Validity	Structural Validity	Hypothesis Testing	Cross Cultural Validity	Criterion Validity
Congenital Ichthyosis	*IQOL*	*13*	*32*	*CTT*	*Poor*	*/*	*/*	*Good*	*Poor*	*Good*	*/*	*N/A*
	*FBI*	*14*	*25*	*CTT*	*Fair*	*Fair*	*/*	*Good*	*Good*	*Fair*	*Good*	*N/A*
Pachyonychia Congenita	*IPCRR*	*15–16*	*30*	*CTT*	*/*	*/*	*/*	*Poor*	*/*	*Poor*	*/*	*N/A*
	*PC QOL*	*17*	*12*	*IRT*	*Good*	*Good*	*Good*	*Excellent*	*Good*	*Good*	*/*	*N/A*
Darier Disease	*DD-QOL*	*18*	*8*	*CTT*	*Poor*	*/*	*/*	*Poor*	*Poor*	*/*	*/*	*N/A*
Neurofibromatosis Type 2	*NFTI-QoL*	*19*	*8*	*CTT*	*Good*	*Poor*	*/*	*Good*	*Good*	*Good*	*/*	*N/A*
HHT	*EQ QOL*	*20*	*13*	*CTT*	*Good*	*Good*	*Good*	*Excellent*	*Good*	*Good*	*/*	*N/A*
	*Psychosocial in HHT*	*21*	*71*	*CTT*	*/*	*/*	*/*	*Poor*	*/*	*/*	*/*	*N/A*
Hereditary Angioedema	*IHAE-QoL*	*22*	*11*	*CTT*	*Good*	*Good*	*Good*	*Good*	*Good*	*Good*	*/*	*N/A*
	*HAE-PRO*	*23*	*18*		*/*	*/*	*/*	*Excellent*	*/*	*/*	*Good*	*N/A*
Epidermolysis Bullosa	*QOLEB*	*12*	*17*	*CTT*	*Good*	*Good*	*Good*	*Excellent*	*Good*	*Good*	*Good*	*N/A*
	*EB-BoD*	*24*	*20*	*CTT*	*Good*	*Good*	*Good*	*Excellent*	*Good*	*Excellent*	*Good*	*N/A*
Fabry Disease	*FSPHPQ*	*25*	*40*	*CTT*	*Good*	*Good*	*Good*	*Excellent*	*Good*	*Good*	*Fair*	*N/A*
Basal Cell Nevus Syndrome	*BCCNS QoL (aBCCdex)*	*26–27*	*26*	*CTT*	*Good*	*Good*	*Good*	*Excellent*	*Good*	*Excellent*	*/*	*N/A*
Peutz Jegher Syndrome	*PJS QoL Questionnaire*	*28*	*31*	*CTT*	*Poor*	*Poor*	*/*	*Fair*	*Good*	*Fair*	*/*	*N/A*

Abbreviations: IQOL (Ichthyosis QoL Questionnaire); FBI (Family Burden in Ichthyosis Questionnarie) IPCRR (International Pachynoychia Congenita Research Registry); DD-QOL (Darier Disease QoL Questionnaire); NFTI-QoL (Neurofibromatosis Type 2 Impact on QoL); HHT (Hereditary Haemorrhagic Telangiectasia); EQ-QOL (Epistaxis-specific QoL Questionnaire); IHAE-QoL (Inherited Angioedema QoL Questionnaire); FSPHPQ (Fabry Specific Pediatric Health and Pain Questionnaire) BCCNS (Basal Cell Carcinoma Nevus Syndrome); PJS (Peutz Jegher Syndrome)

**Table 4:** Critical evaluation of Disease-Specific PROMs using “Quality criteria for measurement properties of health status questionnaires”

Condition	HR-QoL PROM	Study	Content Validity	Internal Consistency	Criterion Validity	Construct Validity	Reproducibility		Responsiveness	Floor and Ceiling Effects	Interpretability	Score (A-D)	Used in Studies?
							Agreement	Reliability					
Congenital Ichthyosis	*IQOL*	*13*	+	+ (α = 0.94)	*N/A*	+	+	*0*	0	0	+ (MIC = 9)	*C*	*No*
	*FBI*	*14*	+	+ (α = 0.89)	*N/A*	?	+	*?*	0	0	+ (MIC = 25)	*B*	*No*
Pachyonychia Congenita	*IPCRR*	*15,16*	–	0	*N/A*	0	0	*0*	0	0	0	*D*	*No*
	*PC QOL*	*17*	+	+	*N/A*	?	?	*+*	0	?	0	*B*	*No*
Darier Disease	*DD-QOL*	*18*	+	0	*N/A*	?	0	*0*	0	0	0	*D*	*No*
Neurofibromatosis Type 2	*NFTI-QoL*	*19*	+	+ (α = 0.87)	*N/A*	+	0	*0*	0	0	?	*B*	*Yes* [2]
HHT	*EQ QOL*	*20*	+	+ (α = 0.95)	*N/A*	?	+	*?*	0	0	+ (MIC = 12)	*B*	*Yes* [1]
	*Psychosocial in HHT*	*21*	–	0	*N/A*	0	0	*0*	0	0	0	*C*	*No*
Hereditary Angioedema	*IHAE-QoL*	*22*	+	-(α = 0.63–0.88)	*N/A*	?	?	*+*	0	0	0	*C*	*No*
	*HAE-PRO*	*23*	+	0	*N/A*	0	0	*0*	0	0	0	*D*	*No*
Epidermolysis Bullosa	*QOLEB*	*12*	+	+ (α = 0.92)	*N/A*	+	+	*?*	0	?	+ (MIC = 6)	*B*	*Yes* [6]
	*EB-BoD*	*24*	+	+ (α = 0.9)	*N/A*	+	?	*+*	0	0	0	*B*	*No*
Fabry Disease	*FSPHPQ*	*25*	+	0	*N/A*	0	?	*?*	0	0	0	*B*	*No*
Basal Cell Nevus Syndrome	*BCCNS QoL (aBCCdex)*	*26,27*	+	+ (α = 0.86–0.93)	*N/A*	+	?	*+*	0	–	?	*C*	*No*
Peutz Jegher Syndrome	*PJS QoL Questionnaire*	*28*	+	–	*N/A*	?	0	*0*	0	0	0	*C*	*No*

Abbreviations: *IQOL* (Ichthyosis QoL Questionnaire), *FBI* (Family Burden in Ichthyosis Questionnarie) *IPCRR* (International Pachyonychia Congenita Research Registry), *DD-QOL* (Darier Disease QoL Questionnaire), *NFTI-QoL* (Neurofibromatosis Type 2 Impact on QoL), *HHT* (Hereditary Haemorrhagic Telangiectasia), *EQ-QOL* (Epistaxis-specific QoL Questionnaire), *IHAE-QoL* (Inherited Angioedema QoL Questionnaire), *FSPHPQ* (Fabry Specific Pediatric Health and Pain Questionnaire) *BCCNS* (Basal Cell Carcinoma Nevus Syndrome), *PJS* (Peutz Jegher Syndrome)

*+ = Positive Rating*? *= Indeterminate Rating - = Negative Rating 0 = No Information available MIC = Minimal Important Change, α = Cronbach’s Alpha*

*(Four degrees of recommendation will be made: A) QoL measurement instrument meets all requirements and is recommended for use; B) QoL measure meets two or more quality items, but performance in all other required quality items is unclear, so that the outcome measure has the potential to be recommended in the future depending on the results of further validation studies; C) QoL measure has low quality in at least one required quality criterion (≥1 rating of “minus”) and therefore is not recommended to be used any more; D) QoL measure has (almost) not been validated. Its performance in all or most relevant quality items is unclear, so that it is not recommended to be used until further validation studies clarify its quality)*

Two disease-specific PROMs exist for QoL evaluation in inherited ichthyoses: The IQoL-32 [13] which is designed for evaluation of HR-QoL in individuals with ichthyoses; and the Family Burden Ichthyosis (FBI) Questionnaire [14] designed for assessment of HR-QoL impact upon those caring for an individual with ichthyosis. To date no interventional trials have utilised these PROM. Assessment of QoL in Pachyonychia congenital (PC) has involved the use of the IPCRR Questionnaire [15, 16], which combines questions pertaining to PC phenotype with HR-QoL evaluation [15]. The IPCRR has not been used thus far to evaluate HR-QoL in interventional trials [15, 16]. The darier disease health related quality of life questionnaire (DD-QOL) has been developed [18] but is yet to be utilised in assessment of therapeutic interventions. Validated HR-QoL PROMs (including the NFTI-QoL) [19] exist for visual and neurological impairment associated with neurofibromatosis (NF) [19]. The NFTI-QoL has been used for measuring longitudinal changes in HR-QoL in NF patients as well as evaluating the impact of auditory brainstem implants [29, 30]. The EQ-QOL is an epistaxis-specific HR-QoL questionnaire (EQ-QOL) [20] for use in hereditary haemorrhagic telangectasia (HHT) and has highlighted the frequency of epistaxis, duration of epistaxis and history of prophylactic embolisation as predictive of HR-QOL impact in HHT via EQQOL scores [20, 21]. It has been used in 1 randomised control trial of iron supplementation in HHT [31] as well as two observational studies [32, 33]. The IHAE-QOL [22] (Impact of Hereditary Angioedema) is a validated disease-specific HR-QOL PROM developed for hereditary angioedema patients [28]. The HAE-PRO [23] is similarly designed for hereditary angioedema patients and is currently undergoing further validation. Both PROMs are yet to be used in observational or interventional studies [22, 23]. In Epidermolysis Bullosa, the QOLEB [12] is a HR-QoL PROM for individuals, whereas the EB-BoD [24] is designed to evaluate the burden of disease upon family members. The QOLEB has been utilised in observational [33–36] as well as interventional studies [37–39]. The fabry-specific FPHPQ [25] BCCNS QoL Questionnaire (aBCCdex) [26, 27] and Peutz-Jeagher Syndrome (PJS) Questionnaire [28] all measure HR-QOL in individuals affected by respective conditions and have not yet been utilised in published interventional studies.

### COSMIN Checklist

#### Classical Test Theory vs Item Response Theory

Fourteen of the fifteen studies identified in this review were developed in line with Classical test theory (CTT). Only the PC-QOL for HR-QOL in pachyonychia congenita was developed in line with item response theory (IRT).

#### Internal Consistency

Eight of the fifteen studies rated ‘Good’ on the COSMIN checklist with 1 study rating fair and 2 ‘Poor’. An additional 3 studies did not have any documentation regarding internal consistency and it was unable to be determined whether this had been calculated. Unidimensional Cronbach’s alpha was calculated in 9 of the 15 studies however the most common downfall leading to a lack of ‘Excellent’ COSMIN rating was the lack of accounting for missing items. Alpha ranged from 0.63 (IHAE-QoL) – 0.95 (EQ-QoL), with eight of the nine studies demonstrating good (0.9 > α > 0.8) or excellent (α > 0.9) [40] with the EQ-QOL demonstrating an borderline α of 0.95, bringing into question whether redundant items exist.

#### Reliability

Seven of the fifteen studies rated ‘Good’ on the COSMIN checklist with regards to reliability, with one study rated ‘fair’ (Family Burden in Ichthyosis (FBI) Questionnaire) and two studies (NFTI-QoL and PJS Questionnaire) rated as ‘Poor’. The fair rating was given as the spearman correlation coefficient was described, but no evidence was presented that no systemic change in the patient’s condition had occurred. The poor ratings were given as no reliability analyses were undertaken in either the NFTI-QoL or PJS Questionnaires.

#### Measurement Error

Less than half of studies assessed contained evaluation of measurement error. All studies that did not include a reliability measurement (IPCRR, DD-QOL, NFTI-Qol, Psychosocial in HHT, HAE-PRO, PJS) automatically did not complete a measurement error analysis due to the common methodology for the two assessments. Studies which presented reliability measurements provided data which enabled the standard error of measurement (SEM) or Limits of Agreement (LOA) to be calculated, resulting in a rating of ‘Good’. No studies provided LOA or smallest detectable change (SDC) statistics.

#### Content Validity

The determination of content validity involves a judgement regarding the relevance and comprehensiveness of items. The most common methods in the studies examined included patient focus groups, opinion of experts in the field. Seven studies scored ‘Excellent’ for content validity with four studies (IQOL, FBI, NFTI-QOL, IHAE-QoL) assessed as ‘Good’. The discriminating factor between ‘Excellent’ and ‘Good’ scores was the inclusion of assessment of the relevance of items to both the patient population and the purpose of the PROM by expert consensus or trial distribution to the study population. The PJS QoL Questionnaire was rated ‘Fair’ as there was insufficient information available that the items comprehensively reflected HR-QoL in this patient cohort. Two studies (IPCRR, Psychosocial in HHT) were assessed as ‘Poor’ as there was no evidence that items were assessed for relevance to HR-QoL.

#### Structural validity

Exploratory or confirmatory factor analysis was undertaken in ten studies with all corresponding measures rated ‘Good’ on the COSMIN checklist. No specific description as to the handling of missing items was available in the text, leading to a ‘Good’ as opposed to ‘Excellent’ rating. The IQOL and the DD-QOL scored ‘Poor’ as no factor analysis was undertaken in these studies. Three studies (IPCRR, Psychosocial in HHT, HAE-PRO) did not provide information on factor analysis, however the HAE-PRO PROM is under ongoing development, with only details of development and content validity published to date.

#### Hypothesis testing

Two studies scored ‘Excellent’ ratings (EB-BOD, BCCNS QoL) with 7 studies rating ‘Good’, 2 ‘Fair’ (FBI, PJS QoL) and the IPCRR rating ‘Poor’. Three studies (DD-QOL, Psychosocial in HHT and HAE-PRO) had no hypothesis testing in the papers examined. The EB-BOD and BCCNS QoL defined clear a priori hypotheses in magnitude and direction, the inclusion of both magnitude and direction of expected correlation distinguishing these from the majority of PROM rating ‘Good’ in this domain. The FBI and PJS -QOL were rated ‘Fair’ as only vague hypotheses regarding the correlation of the PROM with additional PROM (the SF-12 and SF36/CES-D respectively) could be deduced from the text. The IPCRR had no information of comparator PROMs stating only that *‘plantar keratoderma…led to the greatest effect on quality of life’* [16].

#### Cross Cultural Validity

Only five of the fifteen studies undertook cross-cultural validity testing. The IHAE-Qol reported translations available in 13 languages [22] but the methodology of cross-cultural validation was unable to be assessed. The FBI as well as the EB-BoD was translated and validated from French to English [14, 24]. The HAE-PRO was validated for six distinct languages [23], the QOLEB has been validated in Dutch, Spanish and Portuguese [34–36], with the Fabry Specific Health and Pain Questionnaire validated in 8 languages [25]. No studies scored an ‘excellent’ rating as all validations included multiple forward translations but only one backward translation. Also, information regardingfactor analysis and/or differential item functioning after translation was variable. The Fabry Specific Health and Pain Questionnaire was scored ‘Fair’ as insufficient information was available to assess the number and independence of the translations.

#### Responsiveness, Interpretability and Generalisability

None of the identified studies included assessment of responsiveness with a priori hypotheses as required by COSMIN Criteria. Four studies (IQOL, FBI, EQQOL and QOLEB) provided Minimal Important Change (MIC) scores. With regards to generalisability, response rates for the assessed studies ranged from 36.8% (IPCRR) to 98% (EQQOL). The majority of studies were hospital based with additional recruitment from patient support groups. Ethnically, the majority of studies enrolled majority Caucasian populations of middle to high earning income brackets with an equal distribution of gender. The average age of patients ranged from 36.2 (IQOL) to 55.7 years (EQ-QOL).

### Assessment of Measurement Properties

#### Content Validity

In line with assessment using COSMIN criteria, only 2 PROMs were given a negative rating for content validity (IPCRR and Psychosocial in HHT). All other PROMs were given a positive rating with no indeterminate ratings.

### Internal Consistency

Cronbach’s alpha was calculated in 9 of the 15 PROMs as discussed in the COSMIN assessment of internal consistency. 8 Of the 9 PROMs received positive ratings with the IHAE-QoL having a Cronbach’s α below 0.9 (α = 0.63–0.88).

#### Construct Validity

5 positive ratings and 6 indeterminate ratings were given for construct validity. The main deficiencies were the lack of predefined hypotheses, which resulted in all indeterminate ratings being given due to a lack of clarity regarding if conclusions drawn regarding variations in scores between subgroups were based upon a priori hypotheses. No information was available for the remaining 4 PROMs.

#### Reproducibility (Agreement)

Four positive ratings were given for agreement. Five PROMs were given indeterminate ratings as the SDC could be calculated by using the provided SEM, however no measurement of interpretability (MIC) was available for comparison. All four studies which provided the MIC were given positive ratings as the SDC was less than the MIC.

#### Reproducibility (Reliability)

Four positive ratings were given for reproducibility. An additional four were given indeterminate ratings. The majority of studies utilised spearman correlation coefficients for this measurement, which does not take into account systematic differences [9] and hence did not warrant a positive rating.

#### Responsiveness

No information regarding responsiveness was found in any of the studies.

#### Floor and Ceiling Effects

Floor and ceiling effects were only examined in the PCQoL and the BCCNS QoL. The PCQoL stated that some floor effects were found but did not quantify the proportion of items affected. The BCCNS Qol had an effect cut-off of 25% (compared with the standard 15%) and hence was given a negative rating.

#### Interpretability

Four PROMs (IQoL, FBI, EQQol and QOLEB) defined MIC, with the BCCNS QoL stating calculation could not be done due to the poor correlation between the anchor and the PROM [26, 27]. The remainder of PROMs did not provide information on MIC.

### Overall Recommendations

The overall recommendations are presented in Table 4. As criterion validity is inappropriate to assess in HR-QoL PROMs, this criteria was excluded from the development of overall recommendations. No PROMs met all criteria necessary for an ‘A’ recommendation to be given. Seven PROMs were rated ‘B’ (with further clarification required in the properties of agreement, responsiveness and floor and ceiling effects being common themes. A ‘C’ rating was given to five PROMs (IQOL, IHAE-QoL, BCCNS-QoL, Psychosocial in HHT and PJS QoL) due to a negative rating in at least one domain. The remaining three PROMs (HAE-PRO, DD-QOL and IPCRR) scoring a ‘D’ rating, with the majority of properties unable to be rated due to lack of data. All PROMs rated ‘C’ are recommended to no longer be used and all PROMs rated D require re-evaluation subject to further validation studies.

## Discussion

Of the fifteen disease-specific PROM in genodermatoses, none could be recommended for current use, with an additional seven appropriate for use, but only recommended if deficiencies in the domains in Tables 3 and 4 are addressed in future studies.

Deficiencies identified in development included internal consistency (3 ‘Poor’ ratings and 3 PROMS not evaluated), reliability (two ‘Poor’ ratings and 5 PROMs not evaluated) Content validity was the strongest domain evaluated with seven ratings of ‘Excellent’. Cross cultural validity was largely unreported, although, when completed, the standard was generally acceptable. Deficiencies in measurement properties included floor and ceiling effects (1 negative rating and 2 indeterminate ratings) and agreement (9 indeterminate ratings). No data was available for responsiveness. Some confusion was evident in the variable nomenclature of measurement properties and we would suggest adherence to a pre-defined set of defintions (eg COSMIN definitions) for consistency. To maximise generalisability, PROMs require validation in ethnically diverse contexts [41] as well as adolescent and paediatric populations [42]. Tailoring PROMs to age-specific ranges maximises patient understanding, as well as eliminating concern over parental proxy over-reporting of disease burden [43].

The strengths of this review include the evaluation of both the development (using COSMIN criteria) as well as the measurement properties of the identified PROMs. Providing overall recommendations regarding the quality of PROMs in genodermatoses will provide researchers and practicing dermatologists (including those with little knowledge of outcomes research methods) with the evidence to select the most appropriate PROM for assessment of a genodermatosis of interest. The limitations of this review include the late registration of this study with PROSPERO, after the commencement of formal screening of studies. Despite this late registration all methodology for this systematic review were pre-specified prior to the commencement of review. However, the delay in registration may potentially contribute to review bias in study eligibility criteria as defined by the ROBIS [44] Group.

## Conclusion

HR-QoL assessment in genodermatoses with generic PROMs demonstrates deficiencies in content validity and sensitivity. There is a need for a profound paradigm shift in the standard to which HR-QoL PROMs in dermatology are held to account. Of the fifteen PROMs identified in none met current COSMIN standards for recommendation. Valid and reliable disease-specific PROMs do exist however many existing PROMs require additional psychometric analysis. Dermatologists need to be aware of the deficiencies in existing PROMs in order to support high quality data in clinical trials involving genodermatoses.

## Additional files

Additional file 1: Figure S1. Search Strategy. (DOCX 13 kb)

Additional file 2: Figure S2. List of Articles Which Underwent Full Text Screening. (DOCX 22 kb)
