# Supplementary material for: Disease-specific health related quality of life patient reported outcome measures in Genodermatoses: a systematic review and critical evaluation
Source: Orphanet J Rare Dis. 2017 Dec 29;12:189. doi: 10.1186/s13023-017-0739-5 (PMC5747090; doi:10.1186/s13023-017-0739-5)
Supplement: Supplementary file 1 — Search Strategy. (DOCX 13 kb) [file 13023_2017_739_MOESM1_ESM.docx]

| Resources |  |
| --- | --- |
|  | 1. Ovid Medline* (1946-Oct 16 2017) 2. Embase (1980- Oct 16 2017) 3. PsycINFO (1987- Oct 16 2017) 4. Epub ahead of print, in process and other non-indexed citations (1946- Oct 16 2017) |
| Pubmed Search Strategy |  |
|  | Genodermatosis OR Genodermatoses OR Genetic OR Inherited  AND  Quality of life OR treatment outcome OR patient satisfaction OR questionnaire OR outcome OR patient reported outcome measure OR PROM OR score OR index OR instrument OR inventory  AND  Skin Disease |

**Supplementary Figure 1: Search Strategy**

*Ovid Medline: Both American English and British English spelling variations were used
