# Supplementary material for: Disease-specific health related quality of life patient reported outcome measures in Genodermatoses: a systematic review and critical evaluation
Source: Orphanet J Rare Dis. 2017 Dec 29;12:189. doi: 10.1186/s13023-017-0739-5 (PMC5747090; doi:10.1186/s13023-017-0739-5)
Supplement: Supplementary file 2 — List of Articles Which Underwent Full Text Screening. (DOCX 22 kb) [file 13023_2017_739_MOESM2_ESM.docx]

**Supplementary Figure 2**

1. Kirkorian AY, Weitz NA, Tlougn B Morl KD “Evaluation of Wound Care Options in Patients with Recessive Dystrophic Epidermolysis Bullosa: A Costly Neccessity” Pediatric Dermatol 2013; 31(1);33-37
2. Frew JW, Martin LK, Nijsten T, Murrell DF Quality of life evaluation in epidermolysis bullosa (EB) through the development of the QOLEB questionnaire: an EB-specific quality of life instrument *Brit J Dermatol 2009*;161:1323-30
3. J.W. Frew and D.F. Murrell (2010) “Quality of Life Measurements in Epidermolysis Bullosa: Tools for Clinical Research and Patient Care” *Dermatology Clinics* (2010) 28: 185-90
4. Eismann EA, Lucky AW, Cornwall R “Hand function and quality of life in children with Epidermolysis Bullosa” Pediatric Dermatol 2014; 31(2): 176-182
5. Sampogna F, Tabolli S, Di Pietro C, Castigna D, Zambruno D, Abera D “The evaluation of family impact of recessive dystrophic epidermolysis bullosa using the Italian version of the Family Dermatology Life Quality Index” JEADV 2013;27:1151-1155
6. MOrdin M, Clark M, Doward L, William MK “Pediatric patient report outcome assessment: a case study in Epidermolysis Bullosa” Value in health Conference. ISPOR 15^th^ Annual European Congres Berlin, November 2012
7. Katugampola RP, Anstey AV, Finlay AY et al “A management algorithm for congenital erythropoetic porphyria derived from a study of 29 cases” 2012; 167:888-900
8. Frank J, Poblete Guiterrez “Delayed Diagnosis and Dimished quality of life in erythropoeitic protoporphyria: results of a cross sectional study in Sweden 2010 Journal of Internal Medicine 269: 270-274
9. Holme SA, Anstey AV, Finlay AY, Elder GH, Badminton MN “Erythropoetic protoporphyria in the UK: Clinical Features and effect on Quality of Life. British J Dermatol 2006 155; 574-581
10. Milliward LM, Kelly P, Deacon A, Senior V, Peters TJ “Self rated psychosocial consequences and quality of life in the acute porphyrias” J Inh Metab Dis 2001 733-747
11. Kantola I, Hietaharju A, Taurio J, Kananen K, Kantola T, Viikari J “Quality of Life did nto worsen fo 7 years in enzyme-replacement therapy recipients with Fabry Disease” Clinical Therapeutics 34(45) e21
12. Ramaswami U, Stull DE, Parini R, Pintos-Morell G et al “Measuring patient experiences in Fabry disease: Validation of the Fabry specific Pediatric Health and Pain Questionnaire (FPHPQ) Health and Quality of Life Outcomes 2012; 10:116
13. Watt T, Burlina AP, Cazorla C, et al “Agalsidase beta treatment is associated with improved quality of life patients with Fabry disease: Findings from the Fabry registry” Genetics in Medicine 2010 12(11): 703-712
14. Lourenco C, Bonfim D, Coelho J Marques Jr W “When all the joy is gone: the psychological burden of Fabry Disease in Childhood” Concurrent Poster Session ICNC/AOCCN 2012 Abstracts
15. Simsa K, Clarkea V, Hungb G, Martinc D “A new pain assessment tool for Fabry Disease” Genetics and Metabolism 2011; 102(2);S42
16. Nuss SL. WIlsonn ME “Health related quality of life following haemopoetic stem cell transplantation during childhood” 2007 Journal of Pediatric Oncology Nursing 24(2):106-108
17. Gassas A, Raiman J, White L, Schechter T, Clarke J, Doyle J “Long-Term Adaptive Functioning Outcomes of Children with Inherited Metabolic and Genetic Diseases Treated With Hematopoetic Stem Cell Transplantation in a Single Large Pediatric Center: Parent’s Perspective. J pediatr hematol oncol 2011 33;216-220
18. Perry MB, Suwannarat P, Furst GP, Gahl WA Gerber LH “Musculoskeletal findings and Disability in Alkaptonuria
19. Douglas TD, Ramakrishnan U, Singh RH “Longitudinal Quality of Life Analysis in a phenylketonuria cohort provided sapropritein hydrochloride” Health and quality of life outcomes 2013;11:218
20. Gassio R, Gonzales MJ, Colome R et al “healh related quality of life, sleep and behavioural emotional functioning in early treated phenylketonuric adult patients” J Inherited Metbol Diseases 2013 36(supp1); S127
21. Thimm E, Schmidt LE, heldt K, Spiekerkoetter U “Health related quality of life in children and adolescents with phenylketonuria. Unimpaired HRQoL in patients but feared school failure in patients” J Inherited Metabolic Diseases 2013; 36(5) 767-772
22. Bik-Multanowski M, Didycz B, Morzrzymas R, et al “Quality of life in non compliant adults with phenylketonuria after resumption of diet” J Inherited MEtabol Disease 31(supp2);S415-418
23. Carta MG, Mura G, Sorbello O, Farina G, Demelia L “Quality of Life and Psychiatric Symptoms in Wilson’s Disease: The relevance of Bipolar Disorders” Clinical Practice and Epidemiol in Mental Health 2012;8:102-109
24. Petrovic I, Svetel M, Pekmezovic T et al “Quality of life in patients with Wilson’s disease in Serbia
25. Tillmann HL, Wiese M, Braun Y, et al “Quality of Life in Patients with Various Liver Diseases” Journal Viral Hepatitis 2011;18, 252-261
26. Kunin-Batson A, Erickson N, Ahmed A, Yund B, Shapiro E “Quality of Life after treatment for mucopolysaccharidoses” Molecular Genet and Metabolism 2012; 105(2);S42
27. Back M, Muenzer J, Scarpa M “Evaluation of disease severity in mucopolysaccharidoses” J Ped Rehabil med 2010; 3(1);39-46
28. Bergwerk KL, Rabinowitz YS, Falk RE “Quality of life related to visual function in three young adults with mucopolysaccharidoses” The Scientific world Journal 2003 3:922-929
29. Rallis E, Balatsouras DG, Papdakis P, Economou NC, Kaberos A, Korres S “Urbach-Wiethe Disease” Int J Pediat Otorhinolaryngol 2006 1,1-4
30. Berglund B, Bjork E, Women with Ehlers Danlos Syndrome Experience Low Oral Health-Related Quality of Life J Orofacial Pain 2012;26:307-14
31. Hill CL, Baird WO, Walters SJ “Quality of Life in children and adolescents with Osteogenesis Imperfecta: a qualitative interview based study” 2014 Health and Quality of Life Outcomes” 12(1): 54-63
32. Hald J, FOlkestad L, Harslof T, et al “Health Related Quality of Life in Adults with Osteogenesis Imperfecta is impaired by prevalence of multiple fractures” 2012 J Bone and Mineral Research 27 (Abstract)
33. Sousa T, Bompadre V, White KK “Musculoskeletal Functional Outcomes in Children with Osteogenesis Imperfecta: Associations with Disease Severity and Pamidronate Therapy” J Pediatri Orthop 2014;34:118-122
34. Seikaly MG, Kopanati, Salhab N, Waber P, Patterson D, Browne R, Herring JA “Impact of Alendronate on Quality of Life in Children with Osteogenesis Imperfecta” J Pediatric Orthop 2005;25: 786-791
35. Maymi MA, Martin-Garcia RF “Focal Dermal Hypoplasia with Unusual Cutaneous Features” 2007 Pediatric Dermatol 24(4) 387-390
36. Rand-HenriksenS, Johansen H, Semb SO, Geiran O, Stanghelle JK, FInset A “Health Related Quality of Life in Marfan Syndrome: A cross-sectional study of Short Form 36 in 84 Adults with a verified diagnosis 2010 genet Med 12(8):517-524
37. Fuscar-Poli P, Klersy C, Stramesi F, Callegari A, Arbustini E, Politi P “Determinants of Quality of Life in Marfan Syndrome 2008 Psychosomatics 49:243-248
38. Peters KF, Kong F, Hanslo M Biesecker BB”Living with marfan Syndrome III: Qualiy of Life and Repreoductive Planning” Clin genet 2002 62:110-120
39. Finger RP, Fenwick E, Marella M, Issa PC, Scholl HPN, Holz FG, Lamoureux “The relative impact of vision impairment and cardiovascular disease on quality of life: the example of Pseudoxanthoma elasticum” 2011 Health and Quality of Life Outcomes 9:113
40. Turrion AI, Mayan D, Sellas A, Martin=Holguera R “Health Related Quality of Life in Patients with EDS: EDS and Health related quality of life” Annals of Rheumatic diseases 2013; 72
41. Castori M, Camerota F, Celletti C, Grammatico P, Padua L “Quality of Life in the classic and hypermobile types of Ehlers-Danlos Syndrome. 2010 Annals of Neurology 67(1) 145-146
42. Avshallumova L, Mahoney M “Treatment of Erythrokeratoderma Variabilis with isotretinoin” JAAD 2010 68^th^ Annual Meeting of the AAD. 2010 62(3) Sup 1 AB71
43. Shah S, Boen M, Kenner-Bell B, Schwartz M, Rademaker A, Paller AS “Pachyonychia COngenita in Pediatric Patients Natural History Features and Impact” JAMA Dermatol 2014;150(2):146-153
44. Dufrense H, Hadj-Rabia S, Meni C, Sibaud V, Bodemer C, Taieb C “Family burden in inherited ichthyosis: creation of a specific questionnaire” Orphanet Jounral of Rare Diseases 2013;8:28
45. Mazereeuw-Hautier J, Dreyfus I, Barbarot S, et al “Factors influencing Quality of Life in patients with inherited ichthyosis: a qualitative study using focus groups” BJD 012; 166:646-648
46. Kamalpour L, Gammon B, Chen KH et al “Resource Utilization and Quality of Life Assoicated with Congenital Ichthyoses” Pediatric Dermatol 2011 28(5):512-518
47. Ganemo A, Lindholm C, Lindberg M, Sjoden PO, Vahlquist A “Quality of Life in Adults with Congenital Ichthyoses” Journal of Advanced Nursing 44(4):412-419
48. Dreyfus I, Taieb C, Barabarot S, et al “IQoL-32: A New Ichthyosis-specific measure of quality of life” JAMA Dermatol 2013;69(1):82-87
49. Dodiuk-Gad R, Cohen-Barak E, Ziv M et al “Health Related Quality of Life among Darier’s disease patients” JEADV 2013; 27;51-56
50. Fu T, Leachman SA, Wilson NJ, Smith FJD, Schwartz ME, Tang JY “Genotype-Phenotype Correlations among Pachyonychia Congenita Patients with K16 Mutations” Journal Investigative Dermatol 2011;131:1025-1028
51. Alikhan A, Felsten LM, Daly M, Petronic-Rosic V “Vitiligo: A Comprehensive Overview. Part I: Introduction, Epidemiology, quality of life, diagnosis, differential diagnosis, associations, histopathology, etiology and work up” 2011 J Am Acad Dermatol 65;473-491
52. Montoliu L, Molto E, Fernandez A et al “Towards a universal genetic diagnosis of all types of albinism” 2010
53. Cruz-Inigo AE, Ladizinski B, Sethi A “Albinism in Africa: Stigma, Slaughter and Awareness Campaigns” 2011 Dermatol Clin 29;79-87
54. McCune Albright Syndrome (Café Au Lait Spots): Kelly MH, Brillante B, Kushner H, Robey PG, Collins MT “Physical Function is Impaired but quality of life preserved in patients with fibrous dysplasia of bone” 2005 Bone 37;388-394
55. Hornigold RE, Golding JF, Leschziner G et al “The NFTI-QOL: A Disease-Specific Quality of Life Questionnaire for Neurofibromatosis 2” 2012 J Surgical Neurology 73:104-111
56. Vranceanu AM, Merker VL, Park E, Plotkin SR “Quality fo Life among adult patients with neurofibromatosis 1, neurofibromatosis 2 and schwannomatosis: a systematic review of the literature” 2013 J Neurooncol 114: 257-262
57. Lammens CRM, Bleiker EMA, Verhoef S et al “Psychosocial impact of Von-Hippel-Lindau disease: levels and sources of distress” 2010 Clin Genet 77:483-491
58. Pasculli G, Resta F, Guastamacchia E, Di Genaro L, Suppressa P, Sabba C “Health related quality of life in a rare disease: hereditary hemorrhagic telangiectasia (HHT)or Rendu-Osler-Weber disease” 2004 Quality of Life Research 13: 1715-1723
59. Loaec M, Moriniere S, Hitier M, Ferrant O, Plauchu H, Babin E “Psdychosocial quality of life in hereditary haemorrhagic telangiectasia patients 2011 Rhinology 49; 164-167
60. Geirdal AO, Dheyauldeen S, Bachmann-Haridsta d, Heimdal K “Quality of Life in Patients with hereditary Haemorrhagic Telangectasia in Norway: A Population Based Study” 2012 Am j Med Genet Part A 158A:1269-1278
61. Geirdal AO, Dheyauldeen S, Bachmann-Harildstad G, Heimdal K “Living with Hereditary Haemorrhagic Telangectasia: Coping and Psychological Distress – A Cross Sectional Study” Disability and Rehabilitation 2013;35(3) 206-213
62. Ingrand I, Ingrand P, Gilbert-Dussardier B, et al “Altered Quality of life in Rendu-Osler-Weber disease related to recurrent epistaxis. Rhinology 2011 49, 155-162
63. “Changes in Psychosocial Status after lower limb amputation in a patient with Severe Neuropathic pain because of Mafucci’s Syndrome” 2009 37(4) e9-e12
64. Mathias SD, Chren MM, Colwell HH et al “Assessing Health-Related Quality ofLife for Advances Basal Cell Carcinoma and Basal Cell Carcinoma Nevu Syndrome. Development of the First Disease-Specific Patient Reported Outcome Questionnaires” 2014 JAMA Dermatology 150(2): 169-176
65. Shah M, Mavers M, Bree A, Fosko S, Lents N “Quality of Life and Depression assessment in nevoid basal cell carcinoma syndrome’ 2011 Int J Dermatol 50 268-276
66. Kluger N, Letois F, Picot MC, Guillot B, Bessis D “How much disability is caused by folliculofibromas during Birt-Hogg-Dube Syndrome” JEADV 2011; 25: 940-944
67. Woo A, Sadana A, Mauger DT, Baker MJ, Berk T, McGarrity TJ “Psychosocial impact of Peutz-Jaeghers Syndrome” Familal Cancer 2009 8:59-65
68. Van Lier MGF, Mathus-Vliegen EMH, van Leerdam ME, et al “Quality fo Life and Psychological distress in patients with Peutz-Jeghers Syndrome” Clinical Genetics 2010;78:219-226
69. Prior N, Remor E, Perez-Fernandez et al “IHAE-Qol: Specific health related quality of life (HRQoL) questionnaire in hereditary angioedema due to C1 esterase deficiency (HAE-C1INH)” J Allergy Clin Immunol 133(2) e121
70. Dayno J, Miller DP, Hautamaki E et al “Relationship between angioedema attacks and health related quality of life outcomes in patients with Hereditary angioedema (HAE) J Allergy Clin Immunol 133(2) e122
71. Prior N, Ramor E, Perez-Fernandez E “Validation of an international quality of life questionnaire for hereditary angioedema” 2013 Allergy 68 (suppl 97); 105-114
72. Boulliet L, Launay D, Fain O et al “hereditary Angioedema with C1 esteras deficiency: Clinical presentations and quality of life in 193 French Patients” Annals of Allergy Asthma and Immunology 2013 111(4): 290-294
73. Shearer WT, Notaangelo LD, Griffith LM “Treatment of immunodeficiency: Long term outcome and quality of life” 2008 J Allergy Clin Immunol 122 : 1065-1068
74. Sorensqen R, AEtzioni A, Bousifha AA, Zeiger JB “Collaborating to improve quality fo life in Primary Immunodeficiencies: World PI Week 2013. J Clin Immunol 2013 33: 1145-1148
75. Pavlis MB, Rice ZP, Veledar E et al “Quality of life of Cutaneous Disease in Ectodermal Dysplasia” Pediatric Dermatol 2010; 27(3);2060-265
76. Lane MM, Dalton WT, Sherman SA, Bree AF, Czyzewski DI “Psychosocial Functioning and Quality fo Life in Children and Families Affected by AEC Syndrome” Am J Med genet 20098 149A: 1926-1934
